# Research on Automatic Identification and Rating of Ferrite–Pearlite Grain Boundaries Based on Deep Learning

**DOI:** 10.3390/ma16051974

**Published:** 2023-02-28

**Authors:** Xiaolin Zhu, Yuhong Zhu, Cairong Kang, Mingqi Liu, Qiang Yao, Pingze Zhang, Guanxi Huang, Linning Qian, Zhitao Zhang, Zhengjun Yao

**Affiliations:** 1 College of Material Science and Technology, Nanjing University of Aeronautics and Astronautics, Nanjing 211106, China; 2 Jiangsu Product Quality Testing & Inspection Institute, Nanjing 210007, China; 3 Jiangsu Zhongxin Pipe Sci-Tec Co., Ltd., Nanjing 211100, China; 4 Dongying Industrial Product Inspection & Metrology Verification Center, Dongying 257000, China

**Keywords:** grain size, grain boundary, segmentation, classification, intelligent rating, deep learning

## Abstract

Grain size has a significant effect on the mechanical properties of metals. It is very important to accurately rate the grain size number of steels. This paper presents a model for automatic detection and quantitative analysis of the grain size of ferrite–pearlite two-phase microstructure to segment ferrite grain boundaries. In view of the challenging problem of hidden grain boundaries in pearlite microstructure, the number of hidden grain boundaries is inferred by detecting them with the confidence of average grain size. The grain size number is then rated using the three-circle intercept procedure. The results show that grain boundaries can be accurately segmented by using this procedure. According to the rating results of grain size number of four types of ferrite–pearlite two-phase microstructure samples, the accuracy of this procedure is greater than 90%. The grain size rating results deviate from those calculated by experts using the manual intercept procedure by less than Grade 0.5—the allowable detection error specified in the standard. In addition, the detection time is shortened from 30 min of the manual intercept procedure to 2 s. The procedure presented in this paper allows automatic rating of grain size number of ferrite–pearlite microstructure, thereby effectively improving the detection efficiency and reducing the labor intensity.

## 1. Introduction

Grain size is a parameter used to describe the dimensions of grains of polycrystalline materials. It has a significant effect on the properties of metals [1]. When polycrystalline materials are deformed under external force, the dislocation source will continuously release dislocations, which continuously slip inside the crystal. When dislocations reach the grain boundary, they can only continue moving after overcoming the impediment of the grain boundary to the dislocation movement and transfer the deformation from one grain to adjacent ones, and thus yielding the material. Generally, the grain boundaries in polycrystalline materials have large resistance to deformation, and the deformation of a single grain is implicated by adjacent grains, so the strength of polycrystalline materials at room temperature usually increases with the refinement of grains. That is to say, for polycrystalline materials of the same volume, the smaller the grain, the higher the grain boundary content, the more obvious the impediment of grain boundary to dislocation movement, and the higher the yield limit of the material. This is the fine-grained strengthening effect in polycrystalline materials [2,3]. The Hall–Petch equation [4,5] $\sigma_{s}=\sigma_{0}+Kd^{-\frac{1}{2}}$ is usually used to describe the relationship between the grain size and the yield strength. In the equation, $\sigma_{s}$ is the yield strength of the material, the yield strength of single crystal of the material, d the average grain size, K the gradient of H-P as a constant. Therefore, grain size is a technical item that needs to be strictly controlled in metal processing, especially in hot working. The rating procedures for grain size number include comparison procedure, the planimetric (or Jeffries) procedure, and the intercept counting procedure [6]. In the circle intercept procedure, the error caused by deviation from equiaxed grain can be automatically compensated for without excessive additional fields of view. It is a common procedure that gives consideration to both accuracy and efficiency. In the circle intercept procedure, a number of concentric circles are used to form a measuring network in the metallographic image, and appropriate magnification is selected and used to make the grain boundary intersection count between 50 and 100. During the calculation of intersections, it is counted as one intersection if the measuring circle is tangent to the grain boundary and as two intersections if it is significantly overlapped with the confluence of three grains. The grain size number is calculated by measuring the average grain intercept distance:

(1) $$G=6.643856\mathrm{lg}\frac{MP}{L}-3.288$$

where, *G* is the grain size number; *M* is the magnification used; *P* is the grain boundary intersection count; *L* is the intercept length.

At present, the grain size number in the intercept procedure is mainly rated by manual measurement and calculation. Manual rating has obvious disadvantages. First, it has great subjective influence. Only experienced professionals can accurately identify grain boundaries and judge intersections, and human subjectivity during the measurement affects the rating accuracy. Second, it has low efficiency. For a metallographic image, if the intercept procedure is used, it will take at least 30 min for measurement of hundreds of intersections in multiple fields of view and for the subsequent calculation. Third, it affects health. The rating of grain size number is analyzed under a microscope, and long-term work under high-intensity light will affect the health of testing personnel.

In recent years, the continuous development of artificial intelligence technology has achieved remarkable results in various fields. Deep learning, as the leading artificial intelligence technology, has attracted increasing attention and has achieved excellent performance in medical image classification and text recognition [7,8,9,10]. In the field of material tissue analysis, the automatic metallographic analysis of metal materials based on computer vision and machine learning technologies has become the exploring direction of researchers at home and abroad. Zhang [11] et al. used the dual-threshold method and watershed algorithm to segment different metallographic structures. Geng [12] realized threshold segmentation of 2D exponential gray-level entropy through PCA noise reduction based on Gaussian Mixture Model. Zhu [13] proposed an efficient grain boundary division method based on the structured random tree. Patxi [14] et al. reconstructed the austenite grain boundary in martensitic steel using the deep learning algorithm. Wang [15] et al. applied computer vision technology and machine learning to identify the location of fatigue crack initiation in materials. Azimi [16] et al. pixel-wise segmentation based on Full Convolutional Neural Networks (FCNN), using deep learning method to classify and identify the microstructure of mild steel, achieving 93.94% classification accuracy. In Li’s paper [17], the machine learning method of Gradient Boosting Decision Tree was used to recognize the boundaries of a lath bainitic microstructure. The optimized machine learning model achieved greater than 88% recognition accuracy for all boundaries in the bainitic microstructure, where the recall score of PAG boundary was 93%. Han [18] proposed a high-throughput characterization method based on deep learning, rapid acquisition techniques and mathematical statistics to identify, segment and quantify the microstructure of weathering steel. The segmentation accuracies of 89.95% and 90.86% for non-metallic inclusions and pearlite phases, respectively, and the detection time are significantly reduced. Tharindu [19] proposes a computational design framework and uses artificial neural networks (ANNs) for generating and predicting the forging response of preform shapes, respectively. This study predicted the average effective plastic strain response in spatially varying regions of the forging to within ±8% of the ground truth.

**Figure 1:**
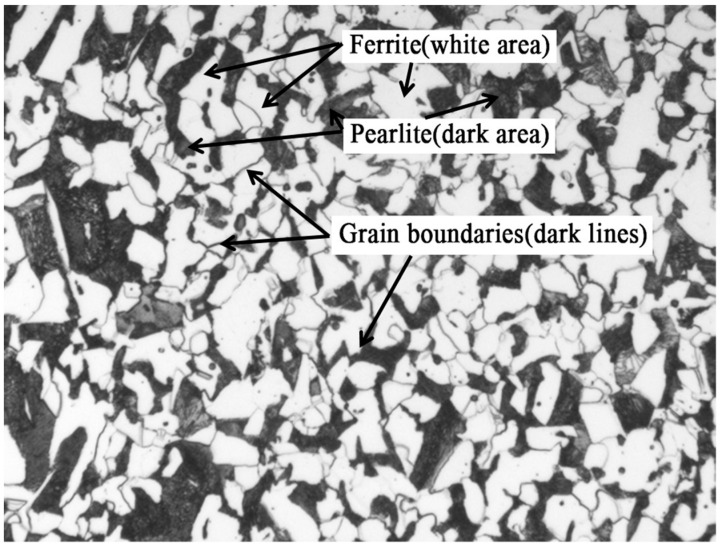
Typical ferrite–pearlite two-phase microstructure.

Currently, scholars have studied the research of using image-processing techniques to identify grain boundaries and calculate grain size [13,14], such as segmentation-based on grayscale thresholding. This technique is an image transformation and analysis based on the gray value of each pixel, which is influenced by factors such as image quality and illumination, and the accuracy of detection is not high. In contrast, using deep learning techniques, the model can be trained and iterated using a large number of calibration samples, and the features of different types of grain boundaries can be extracted, thus achieving accurate segmentation of grain boundaries and accurate calculation of grain size. In this paper, a sample of ferrite–pearlite microstructure (see Figure 1) is used as an example to detect grain boundaries and thus calculate their grain size by using a U-Net-based image segmentation model. These two phases are the most common microstructures in steels. A large number of medium and low-carbon hot-rolled, annealed or normalized steels are ferrite–pearlite microstructures, so it is necessary to study the automatic rating of grain size number of this two-phase microstructure.

There are four facts relevant to the grain size testing of ferrite–pearlite in steel mills. (1) Almost all the steel mills use optical microscopes when testing the grain size. (2) Under the optical microscope, the grain boundary in the ferrite has obvious contrast difference from the grain inside, so such grain boundaries can be easily identified with human eyes. However, for pearlite, due to low resolution, the boundaries of pearlite colonies and the slices of ferrite–cementite cannot be seen. Hence, the pearlite grain size (the size of pearlite colonies) cannot be tested under the optical microscope. (3) Therefore, when the steel mills test the actual grain size of ferrite–pearlitic steel, almost all of them test only the grain size of the ferrite, while the pearlite part is ignored, and finally the grain size of the ferrite part is taken as the overall grain size of the sample. (4) The difficulty of testing the grain size using this method (ferrite only) is different for steels with different carbon contents. For steel with less carbon content, the grain size of ferrite can be easily tested directly because ferrite dominates in the sample and the whole sample indicates a great ferrite region with very little pearlite. However, for steel with high carbon content, since pearlite is randomly and approximately uniformly distributed throughout the sample and there is no large pure ferrite area, it is difficult to directly test the grain size of ferrite. In such cases, the presence of pearlite will significantly interfere with the efficiency and accuracy of ferrite grain size testing, and the efficiency of manual calculation of the grain size is extremely low.

Based on the four facts above, when evaluating the grain size of ferrite–pearlite steels by testing the ferrite grain size, in order to solve the interference of pearlite regions to the testing process and results of ferrite, and to improve the testing efficiency, we propose a new and simple method based on artificial intelligence techniques. Since the steel mills ignore the pearlite and consider the grain size of ferrite as the overall grain size of the sample. Then, we assume that all the pearlite areas are ferrite. At this point, the exact size of grains in the pearlite (considered as ferrite) region is unknown, but it can be predicted according to the confidence of the average grain size of ferrite (actual ferrite) using the model (the grain boundaries predicted by the model, which are not real but assumed by the model, are called “hidden grain boundaries”). In this way, we, (1) identify and test the grain boundaries in ferrite, (2) treat pearlite as ferrite, and use our model to predict the “hidden grain boundaries” inside it, and (3) count and calculate the intersections in the actual grain boundaries in ferrite and the predicted “hidden grain boundaries” in pearlite using the three-circle intercept procedure. Then, statistical calculation is made to achieve automatic rating of grain size number.

## 2. Model and Method

Image segmentation is the first step and also the most important and difficult part of image analysis. Image segmentation is the process of partitioning an image into several disjoint parts by grayscale, color, spatial texture or other characteristics [20], so that these characteristics show consistent differences in adjacent parts, thus achieving the purpose of separating the target from the image. Traditional image segmentation is often carried out with the knowledge of digital image processing, topology, mathematics, etc. Compared with the deep learning-based method, traditional methods have poor ability to deal with impurities, patterns, hidden boundaries, etc. Since the texture of metallographic images is complex [21], and it is difficult and costly to obtain training sets of metallographic images, the image segmentation algorithm of U-Net neural network is used in this paper to extract grain boundaries.

U-Net is a variant of fully convolutional neural network (FCN) [22]. Its architecture is in the shape of a “U”, hence the name. This network consists of two parts: the contracting path and the expanding path. The contracting path is generally used to capture the context information in the image, and the expanding path is used to enable precise localization of the parts to be separated from the image. U-Net is an improved model based on FCN, and it can be trained by using small sample datasets to achieve good segmentation effect through data augmentation. It is especially suitable for application scenarios with high cost and great difficulty in sample collection and calibration, such as metallographic image analysis.

**Figure 2:**
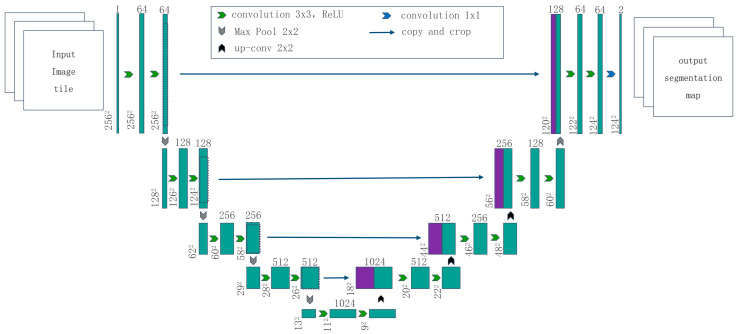
U-Net architecture.

In the contracting path of U-Net, each contraction consists of a 2 × 2 max pooling layer (with stride 2) and two 3 × 3 unpadded convolutional layers. Each unpadded convolutional layer is followed by a ReLU activation parameter for downsampling of original data. At each downsampling step, the number of feature channels is doubled. In the deconvolution of the expanding path, every step consists of a 2 × 2 unpadded convolutional layer (also followed by the activation parameter ReLU) and two sets of 3 × 3 unpadded convolutional layers. At the same time, each deconvolution is followed by an addition of the feature map cropped from the corresponding contracting path (clipped to maintain the same shape). The final layer of the network is a 1 × 1 unpadded convolutional layer that is used to map the 64-channel feature vector to the desired number of classes. Finally, the U-Net used in this paper has 23 layers in total. The advantage of U-Net is that it has no requirement on the shape and size of the input image, especially when extremely large images are processed. Figure 2 illustrates the U-Net architecture used in this paper.

In this paper, cross entropy is used as the loss function of image segmentation, and its principle is as follows:

(2) $$H\left(y^{\left(i\right)}。{\hat{y}}^{\left(i\right)}=-\sum_{j=1}^{q}y_{j}^{\left(i\right)}\log{\hat{y}}_{j}^{\left(i\right)}\right)$$

where, $y_{j}^{\left(i\right)}$ with a subscript is an element equal to either 0 or 1 in the vector $y^{\left(i\right)}$. In the vector $y^{\left(i\right)}$, only the $y{\left(i\right)}^{th}$ element, $y_{y\left(i\right)}^{\left(i\right)}$, equals 1, and all the rest elements equal 0. Therefore,$\;H\left(y^{\left(i\right)}。{\hat{y}}^{\left(i\right)}=-\log{\hat{y}}_{y\left(i\right)}^{\left(i\right)}\right)$. That is, cross entropy focuses only on the prediction of correct classes. If the value of cross entropy is large enough, it indicates that there is no problem with the classification.

Assuming the number of samples in the training dataset is n, the cross-entropy loss function is defined as:

(3) $$l\left(\Theta \right)=\frac{1}{n}\sum_{i=1}^{n}H\left(y^{\left(i\right)}。{\hat{y}}^{\left(i\right)}\right)$$

where Θ is the model parameter. Similarly, if each sample has only one label, the cross-entropy loss can be expressed as $l\left(\Theta \right)=-\frac{1}{n}\sum_{i=1}^{n}log{\hat{y}}_{y\left(i\right)}^{\left(i\right)}$. From another perspective, we know that minimizing l(Θ) is equivalent to maximizing $\exp\left(-nl\left(\Theta \right)\right)=\prod_{i=1}^{n}{\hat{y}}_{y\left(i\right)}^{\left(i\right)}$, that is, minimizing the cross-entropy loss function is equivalent to maximizing the joint prediction probability for all label classes in the training dataset.

The two feature vectors output by U-Net are the category label vector $\;L=\left(L_{1},\cdots ,L_{\eta }\right)$ and the correlation confidence degree $F=\left(F_{1},\cdots ,F_{\eta }\right)$, where $L_{i},F_{i}=F\left(L_{i}\right)$ are the category label of the ith pixel and the corresponding confidence degree respectively. $L_{i}\in \{c_{1},c_{2},\cdots ,c_{\tau }|c_{j}\in N\}$ is a class label; $0\leq F_{i}\leq 1$ is the confidence value for the ith pixel classified as $l_{i}$. In the above definition, 1≤i≤η, η is the maximum order value obtained by sequencing the training image pixels in the way of the first method.

## 3. Experiment

### 3.1. Dataset

A total of 200 metallographic samples with a diameter of 10 mm were prepared with hot-rolled ribbed bars as the test samples. After grinding, polishing and corrosion, the samples showed clear ferrite–pearlite two-phase microstructures and ferrite grain boundaries.

**Figure 3:**
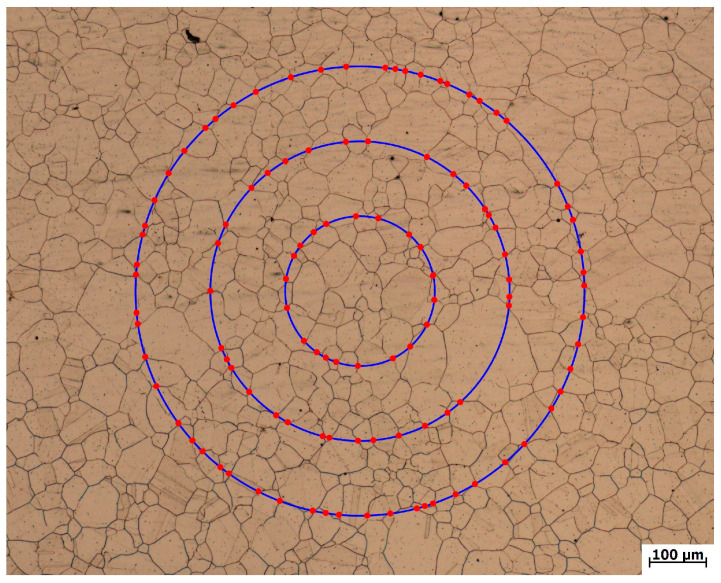
Example of Three-Circle Intercept Calibration.

The LabelMe platform was used to distribute grain size images for experts, and the circle intercept procedure was used for the measurement and statistics of intersections and the calculation of grain size. It should be noted that hidden boundaries do not need to be marked manually, except for visible contour lines. Figure 3 shows an example of a single grain size image and its three-circle intercept calibration. In this paper, 2000 valid grain size images and their annotation files were finally collected, with the image size of 2048 × 1536. The grain sizes of these images were calibrated and classified by experts and then imported into the dataset.

### 3.2. Data Augmentation

For deep learning, the size of the dataset has a great impact on the effect of the model [23]. A too-small dataset will lead to overfitting of the model and thus inaccurate prediction results. The dataset used in this paper only included a few metallographic image samples, making it difficult to obtain good results. Therefore, the existing data were augmented to expand the dataset. For this dataset, the data were mainly augmented by random rotation, flipping and scaling, and random erasing. Data augmentation mainly serves two purposes: (1) to increase the data volume for training, so that when the data volume is large, there will be many characteristics, resulting in an improvement in the generalization ability of the model; (2) to eliminate the impact of noise data that increases with the volume of the effective data, so as to improve the robustness of the model. Taking random erasing as an example, the model sometimes shows high consistency with the training data, but does not work very well in data verification, that is to say, the model is overfitting. An important factor to image overfitting is that the training data samples are not rich enough. For example, in actual metallographic images, there may be overexposure, underexposure, ambiguity, occlusion of some areas, etc. If we want to improve the prediction and recognition ability in respect of such images and enhance the generalization ability and robustness of the model, it is necessary to add similar forms of data input during training. This is the function of random erasing.

Random rotation and scaling mean randomly rotating the training image by random angle or scaling by random magnification for data augmentation. For a training sample, the augmented sample obtained by image flipping and image rotation can enable the model to learn features that are not deformed by rotation during training, while the augmented sample obtained by image scaling can better enable the model to realize multi-scale training. Let (x,y) be the pixel coordinates of the original image, $\left(x_{1},y_{1}\right)$ be the pixel coordinates after rotation, $\left(x_{2},y_{2}\right)$ be the pixel coordinates after scaling, θ be the rotation angle, (a,b) be the rotation center, *(left,top)* be the coordinates of the upper left corner of the rotated image,${\;s}_{x}$ and $s_{y}$ be the scaling factors. The rotation and scaling formulas are as follows:

$$\left[\begin{array}{ccc} x_{1} & y_{1} & 1 \end{array}]=[\begin{array}{ccc} x & y & 1 \end{array}\right]\left[\begin{array}{ccc} 1 & 0 & 0\\ 0 & -1 & 0\\ -a & b & 1 \end{array}\right]\left[\begin{array}{ccc} cos\theta & -sin\theta & 0\\ sin\theta & cos\theta & 0\\ 0 & 0 & 1 \end{array}\right]\left[\begin{array}{ccc} 1 & 0 & 0\\ 0 & -1 & 0\\ left & top & 1 \end{array}\right]\;$$

$$\left[\begin{array}{ccc} x_{2} & y_{2} & 1 \end{array}]=[\begin{array}{ccc} x & y & 1 \end{array}\right]\left[\begin{array}{ccc} s_{x} & 0 & 0\\ 0 & s_{y} & 0\\ 0 & 0 & 1 \end{array}\right]$$

**Figure 4:**
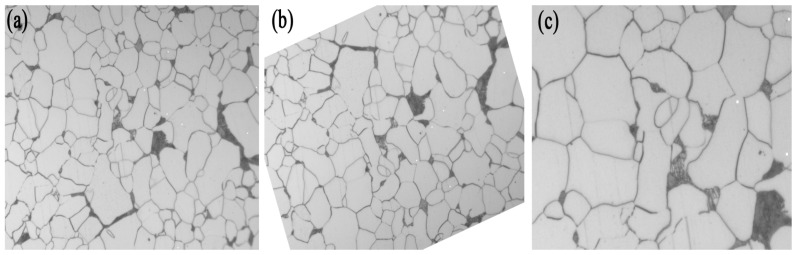
Random rotation and scaling images (Original (**a**), Rotated (**b**), Enlarged (**c**)).

The results are shown in Figure 4.

**Figure 5:**
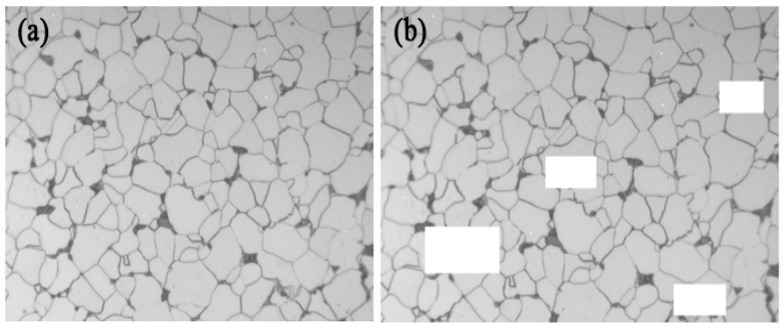
Random erasing images (Original (**a**), Erased (**b**)).

Random erasing is a new data measurement technique, which erases the pixels with random values in the randomly selected rectangular areas in training, so as to obtain more occlusions or erase pixel values and images with different areas for data enhancement. This technique can reduce the risk of fitting and make the model robust to occlusion and erasure. The results are shown in Figure 5.

### 3.3. Algorithm Steps

The algorithm used in this paper falls into five modules: metallographic image acquisition module, contour extraction network module, visible intersection statistics module, hidden intersection statistics module and automatic rating module.

The image acquisition module measures the metallographic samples and calculates their grain size number by the conventional three-circle intercept procedure through experts of equivalent level, labels the images with the LabelMe v5.1.1 software and uploads them to this dataset for image acquisition. The module contains 2000 effective images of grain size numbers and their labeling files, which are used as the training set of the contour extraction network module.

**Figure 6:**
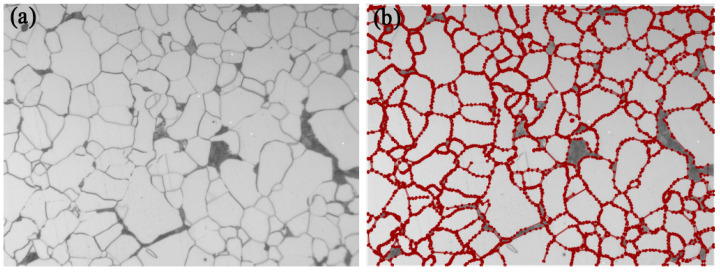
Original image (**a**) and Labeled image (**b**).

**Figure 7:**
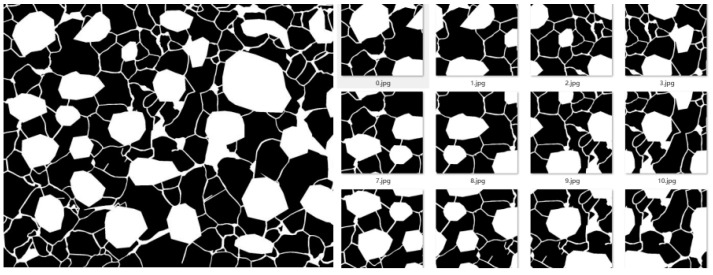
Original Labeled image (**Left**) and Segment image (**Right**).

The contour extraction network module is mainly for image preprocessing and grain boundaries contour extraction. One of the most critical steps is the training of the model, which is specifically divided into the following substeps: (1) Calibrate the original data of image size 2048 × 1536 with LabelMe, mark the visible boundary and fill the prolongable boundary. Name the images with id_magnification used_level.png (See Figure 6). (2) The Labeled image of Figure 6b is binarized and then a random mask is applied to form Figure 7a. Segment the labeled images into images of size 256 × 256 with a step size of 128 and segment the labels in the same way (See Figure 7). (3) Input the dataset into U-Net for training, use the PyTorch framework for model building and training, and save the trained model files.

**Figure 8:**
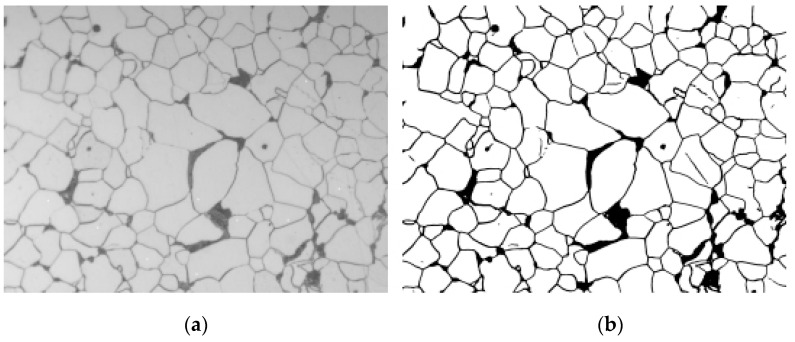
Grain-boundary contours identified by the model. Original image (**a**) and image of model predictions (**b**).

Start the test after the model is trained. Enter a metallographic image of size 2048 × 1536. The program first segments the image, runs the contour extraction model for model extraction, and finally splices the segmented images, and processes the overlapping part with the mean value. In this way, a complete contour extraction image will be obtained. Both model training and prediction are carried out in an end-to-end manner. Extraction of contours by sections greatly reduces the complexity of model training and improves the running speed of the model. The formula for dealing with overlapping parts is as follows:

$$x_{avg}=average\left(x_{1},x_{2}\cdots x_{n}\right)\;,\;x_{n}\in \left\{0,1\right\}$$

$$x_{new}=\{\begin{array}{c} 1\;,x_{avg}>\alpha \\ 0,x_{avg}\leq \alpha \end{array},\alpha \in \left(0,1\right)$$

where, n represents the number of overlaps, $x_{n}$ represents the value of the nth overlap prediction, and the value is 0 or 1,0 represents that this pixel is not predicted to be the contour, 1 represents that this pixel is predicted to be the contour, average is the mean operation, the result is $x_{avg}$, α is the classification threshold, and the value in this paper is 0.8, $x_{new}$ represents the type of the overlap region after processing. Contour extraction results are as Figure 8:

As for the visible intersection count statistics module, the binary image of edge contour output by the contour extraction network is input, and based on the three-circle truncation method, the edge contour line is judged to intersect the circle, and the position of the pixel where the intersection point is recorded, so as to conduct preliminary statistics of the truncation point, so the number of visible cut-off points P is calculated.

As for the hidden intersection count statistics module, the binary image of the grain boundary contour output by the contour extraction network is input, and the intersection count of the pearlite area is estimated based on confidence. The calculation of grain size number is a description of the overall ferrite properties. It may be assumed that the size of each grain boundary in a ferrite section is approximately normally distributed after the hidden boundary is delineated. The specific steps are as follows: After the grain boundary segmentation of the first part, we obtain a set of grain boundary sizes $X=\left\{x1,x2,x3..xn\right\},\;x_{n}\;\mathrm{represents}$ the Manhattan distance between two adjacent grain boundaries. For some steel, we solve $\bar{X}=Averge\;\{x_{i}|x_{a}<x_{i}<x_{b}\}$, where $x_{a},x_{b}$ meet $Pr(x_{a}<\mu <x_{b})=\alpha$, which means the calculated average of the confidence interval of the distribution serves as the statistical magnitude describing the overall grain size number of the steel. α varies in different types of ferrites (with different carbon contents), and parameters need to be adjusted for optimization. After computing, $x_{n}$ contains hidden grain boundary cutoff points $x_{new}$ *(*$x_{n}\in \{x_{i}|x_{b}<x_{i},i=0,1\cdots n\}$*)*, finally, present $x_{new}=x÷\bar{x}+1$ to calculate processed grain boundary points (including the number of hidden grain boundary).

The automatic rating module accumulates the visible and hidden intersection count to obtain the final intersection count and calculates the grain size number by Formula (1).

**Figure 9:**
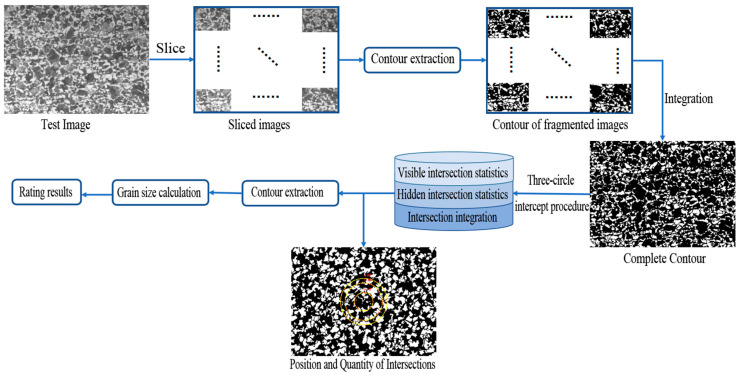
Model architecture.

The model architecture is shown in Figure 9. Capture the target field of view to obtain the image to be rated as the input of the system. Slice the image and input the sliced images into the contour extraction network module for processing, and then integrate them to obtain the contour extraction image. Input the obtained contour into the visible intersection statistics module and the hidden intersection statistics module respectively for calculation. Then, input the results into the automatic rating module for processing to obtain the final rating results and the labeling results by the three-circle intercept procedure.

## 4. Results and Discussion

The training datasets in this paper are all about hot rolled ribbed steel bar samples (HRB400). A trained model is used to test the grain sizes of four different grades of steel, namely SWRCH6A (with a carbon content of 0.05%, close to that of industrial pure iron), HRB400E (with a carbon content ≤ 0.23%), 35K (with a carbon content of 0.32%) and S45C (with a carbon content of 0.45%). With different chemical compositions, they are all of a ferrite–pearlite microstructure. The two-phase ratio of samples with different carbon contents is different. Generally, the higher the carbon content, the higher the pearlite ratio.

### 4.1. Grain Boundary Segmentation Results

The original image size of the datasets used in this paper is 2048 × 1536, and the sub-images of 256 × 256 are cut out with a step size of 128, which fall into the training dataset, verification dataset and test dataset, accounting for 70%, 20% and 10% of the total data respectively. On top of that, the grain size number of three types of steels for which no training data collected are tested.

**Figure 10:**
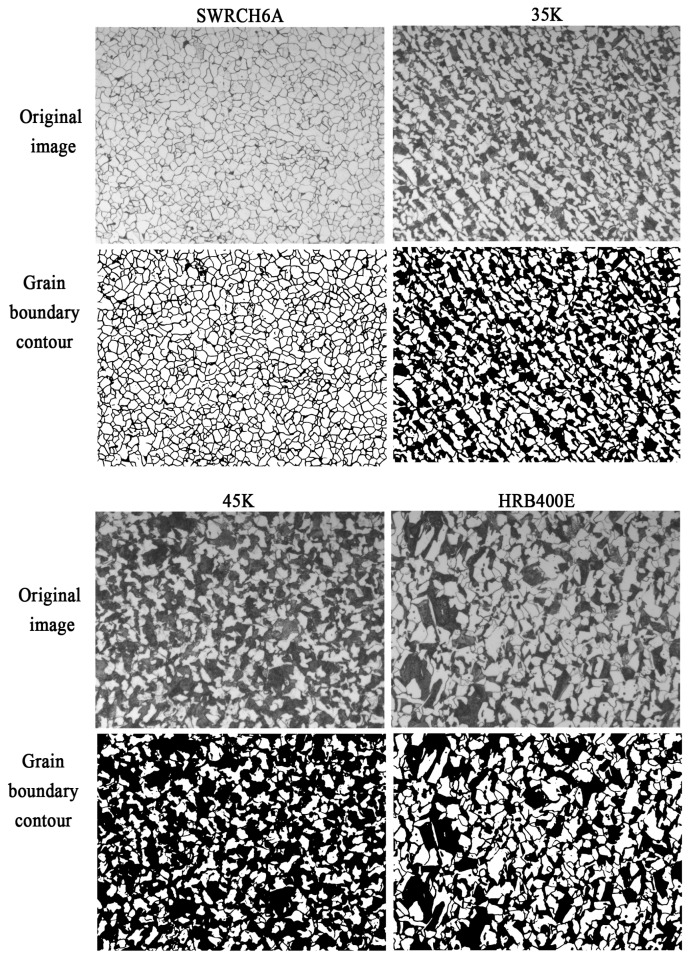
Grain-boundaries segmentation results.

During model training, the authors adjust the number of iteration rounds, learning rate, batch size and other parameters. The results of grain boundary segmentation of various types of steels are shown in Figure 10. It can be seen that the images are well segmented and spliced, showing no obvious splicing joints compared with the original. In different sample images, the grain boundaries inside the ferrite and the ferrite–pearlite interface are all correctly identified and segmented into grain boundary contours as a whole. The pearlite does not show internal grain boundaries, it is treated as a grayscale part as a whole, and its internal hidden grain boundaries are predicted as ferrite.

### 4.2. Rating of Grain Size Number by Circle Intercept Procedure

**Figure 11:**
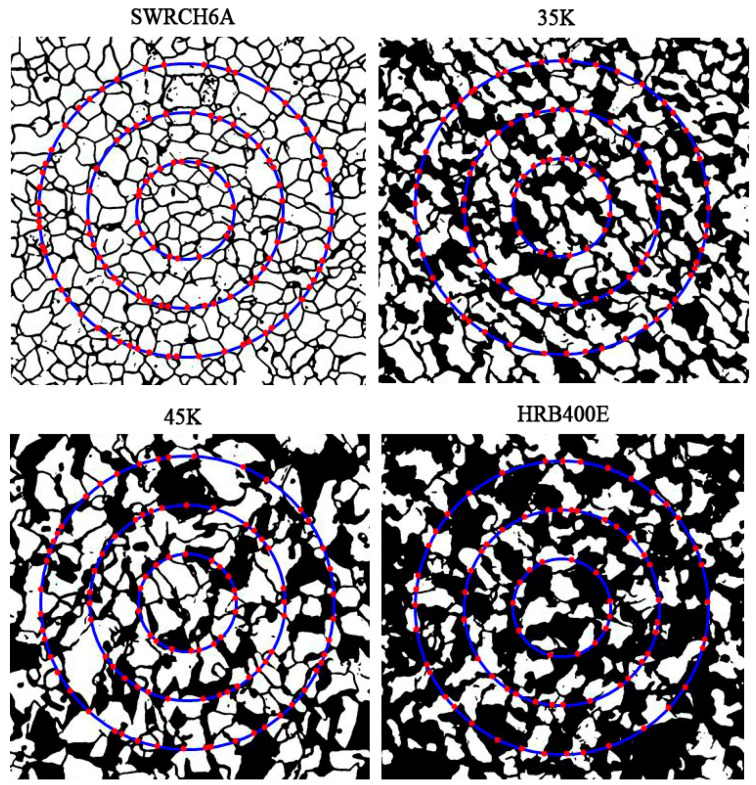
Processing results of four types of steels.

A three-circle intercept procedure is used to count and calculate the grain boundary intersections in the segmented grain boundary images. Three concentric, isometric circles with a total perimeter of 500 mm are used to form a measurement grid, and the point of intersection between the grid and the grain boundary is the intersection. The number of visible intersections in the ferrite microstructure is obtained by counting the binary images of the edge contour with the three-circle intercept procedure. The intersections of the four materials are shown in Figure 11. It can be seen that the intersections of ferrite grain boundaries among the four materials are accurate, and the intersection areas of circumference and ferrite grain boundaries and ferrite–pearlite boundaries are identified as intersections.

In the end, the automatic rating module integrates the visible intersections and hidden intersections to output the rating results and the labeled image of the three-circle intercept procedure. Then, calculate the average grain size number of the samples according to Formula (1). The rating results of the grain size number of the four samples are shown in Table 1. Compared with the manual rating results of experts, the average error is lower than Grade 0.5, meeting the requirements of the allowable error in the detection standard.

The average error and accuracy are defined as follows:

$$e_{i,j}=\left|s_{i,j}-r_{i,j}\right|$$

where i represents the sample number and j represents the sample category. $e_{i,j}$ represents the error of the ith sample of class j*;* $s_{i,j}$ represents the manual rating result of the i*th* sample of class j samples; $r_{i,j}$ represents the model rating result of the ith sample of class j samples.

$$\bar{e_{j}}=\frac{\sum_{i=1}^{n_{j}}e_{i,j}}{n_{j}}$$

$\bar{e_{j}}$ is the average error of the class j. $n_{j}$ represents the total number of class-j samples.

$$A_{j}=\frac{n_{0.5,j}}{n_{j}}$$

$A_{j}$ represents the accuracy of class-j samples and $n_{0.5,j}$ represents the number of samples whose error is less than 0.5.

This paper is based on the model training, prediction and data processing of metallographic images, which inevitably relies on the quality of images. An image will be more representative in characteristics if it has fewer interference items and richer data, and as a result, the model training effect will be better. The original metallographs do have such defects as scratches, foreign contaminants and holes, and they all are interference items for the training and prediction of the deep learning model. In this paper, two methods are mainly used to eliminate the negative impact of these interference items on the model as far as possible. Method 1: accurate data calibration. Scratches, foreign contaminants and holes are different from grain boundaries in form. For example, scratches are mainly long straight lines, while foreign contaminants and holes are mostly points rather than curves. They are obviously different from the grain boundaries that we need to identify. In the preliminary data calibration work, we arranged senior metallographic analysis experts to identify and calibrate the grain boundaries and would not mark these interference items as inclusions. The accurate calibration ensures that, in subsequent training and identification, the model algorithm is unlikely to identify these interference items in the verification sample as grain boundaries. Method 2: Identification through the algorithm. As mentioned earlier, interference items differ from grain boundaries in size, grayscale or shape, and we can avoid misjudgment as far as possible by identifying these differences through the algorithm. For example, in terms of size, grain boundaries are curve segments, so we can directly use the algorithm to delete particles whose length and width are less than a certain value (e.g., 3 μm). In terms of grayscale, the grain boundaries are gray or nearly black. Items with lighter or darker colors are generally foreign contaminants, holes, corrosive water stains, or other interference items. We set a grayscale threshold in the algorithm, and this can also exclude interference items to some extent.

This testing process is an approximate, simple and fast method, suitable for mass production inspection in factories, and cannot be used for accurate detection of grain size. If the true and accurate grain size of the sample is required, it is also necessary to detect the grain size of pearlite (the size of pearlite colonies) besides that of ferrite. In this case, it is necessary to use a scanning electron microscope to obtain a metallographic image with higher resolution, which can clearly display the boundaries of pearlite colonies, and thus the grain size of pearlite can be tested. As we know, the pearlite microstructure is in colonies due to different orientations of ferrite–cementite lamellae, and the boundaries between microstructures of different orientations can be regarded as the grain boundaries of pearlite colonies. Unlike solid boundaries such as ferrite grain boundaries, such boundaries are not solid boundaries formed by lines or specific grayscales or colors, but virtual boundaries formed only due to different orientations of lamellae. The areas on both sides of such virtual boundaries have no difference, except the difference in the orientations of lamellae. Unlike the hidden boundary described earlier as well, this virtual boundary is objectively present and is not visible to the human eyes simply because of the low resolution in the optical images. Such boundaries are easy to identify for man, but difficult for computer. We are studying this aspect and have achieved good results, which will be presented in the next paper.

## 5. Conclusions

This paper presents a model for automatic detection and quantitative analysis of the grain size number of ferrite–pearlite two-phase microstructure. A U-Net neural network model is designed to segment ferrite grain boundaries. In view of the hidden grain boundaries in pearlite microstructure, the number of hidden grain boundaries can be inferred based on the confidence of average grain size. The grain size number is rated using the three-circle intercept procedure. The results show that the accuracy of this procedure is greater than 90% in the grain boundary segmentation for the four types of ferrite–pearlite two-phase microstructure samples. All of these results deviate from those calculated by experts using the manual intercept procedure by less than Grade 0.5, the allowable detection error specified in the standard. In addition, the detection time is shortened to 2 s from 30 min consumed in the manual intercept procedure, greatly improving the detection efficiency and reducing labor intensity. Accordingly, the method proposed in this paper can be effectively used for the automatic rating of grain size number of ferrite–pearlite microstructure. The results obtained can be applied in steelworks laboratories to control the grain size of rolled ferrite–pearlitic steel.

## Figures and Tables

**Table 1:** Detection Results of Various Types of Steel.

Material	Average Error	Accuracy Rate
SWRCH6A	0.3064	96.67%
HRB400E	0.3160	90.00%
35K	0.2843	96.70%
45K	0.2897	93.33%

## Data Availability

The data presented in this study are available on request from the corresponding author.
